# The Role of Tyrosine Phosphorylation of Protein Kinase C Delta in Infection and Inflammation

**DOI:** 10.3390/ijms20061498

**Published:** 2019-03-26

**Authors:** Qingliang Yang, Jordan C. Langston, Yuan Tang, Mohammad F. Kiani, Laurie E. Kilpatrick

**Affiliations:** 1 Department of Mechanical Engineering, College of Engineering, Temple University, Philadelphia, PA 19122, USA; tug44932@temple.edu (Q.Y.); tud19329@temple.edu (Y.T.); mkiani@temple.edu (M.F.K.); 2 Department of Bioengineering, College of Engineering, Temple University, Philadelphia, PA 19122, USA; tuj27061@temple.edu; 3 Department of Radiation Oncology, Lewis Katz School of Medicine, Temple University, Philadelphia, PA 19140, USA; 4 Center for Inflammation, Clinical and Translational Lung Research, Department of Thoracic Medicine and Surgery, Lewis Katz School of Medicine, Temple University, Philadelphia, PA 19140, USA

**Keywords:** PKC, PKCδ, phosphorylation, microfluidics, inflammation, sepsis

## Abstract

Protein Kinase C (PKC) is a family composed of phospholipid-dependent serine/threonine kinases that are master regulators of inflammatory signaling. The activity of different PKCs is context-sensitive and these kinases can be positive or negative regulators of signaling pathways. The delta isoform (PKCδ) is a critical regulator of the inflammatory response in cancer, diabetes, ischemic heart disease, and neurodegenerative diseases. Recent studies implicate PKCδ as an important regulator of the inflammatory response in sepsis. PKCδ, unlike other members of the PKC family, is unique in its regulation by tyrosine phosphorylation, activation mechanisms, and multiple subcellular targets. Inhibition of PKCδ may offer a unique therapeutic approach in sepsis by targeting neutrophil-endothelial cell interactions. In this review, we will describe the overall structure and function of PKCs, with a focus on the specific phosphorylation sites of PKCδ that determine its critical role in cell signaling in inflammatory diseases such as sepsis. Current genetic and pharmacological tools, as well as in vivo models, that are used to examine the role of PKCδ in inflammation and sepsis are presented and the current state of emerging tools such as microfluidic assays in these studies is described.

## 1. Protein Kinase C (PKC) Superfamily 

Protein Kinase C (PKC) was first identified by Nishizuka and coworkers in 1977 and is now known to be composed of a family of phospholipid-dependent serine/threonine kinases [1]. PKC isoforms (PKCs) are involved in numerous signal transduction pathways and are implicated in the regulation of numerous cellular functions [2,3,4]. These kinases are composed of a highly conserved catalytic domain (C-terminus) and a regulatory domain (N-terminus) that demonstrates considerable variability across family members [2]. Based on structural elements and cofactor requirements, mammalian PKCs are classified into four broad categories comprising classical PKCs (cPKCs: α, β-I, β-II, and γ isoforms), novel PKCs (nPKCs: δ, ε, η, and θ isoforms), atypical PKCs (aPKCs: ι and ζ isoforms), and PKC-related kinases (PRKs 1–3) [2,3,4,5,6,7]. Calcium (Ca^2+^) and the lipid second messenger diacylglycerol (DAG) are required for cPKCs activation. DAG, but not (Ca^2+^), activates the nPKCs. The aPKCs do not require Ca^2+^ or DAG for activation, but are sensitive to other lipid second messengers such as phosphatidylserine (PS) [8]. The activity of different PKCs is context-sensitive and these kinases can be positive or negative regulators of signaling pathways. This contextual dependency of the PKC function often makes it difficult to determine the precise roles of PKCs in normal and aberrant cellular processes [8,9]. Increased activity of several PKCs has been implicated in multiple diseases, including inflammation, sepsis, and cancer [5,9]. 

PKCδ is a unique nPKC that plays a significant role in several diseases, including cancer, diabetes, ischemic heart disease, and neurodegenerative diseases [10,11,12,13,14,15,16,17,18,19]. Recent studies from our research group and others have shown that PKCδ is also a critical regulator of the inflammatory response in sepsis [8,19,20,21,22,23,24,25,26]. While a role for PKCδ in sepsis is established, less is known about how PKCδ is activated during the inflammatory response. PKCδ, unlike other members of the PKC family, is unique in its regulation by tyrosine phosphorylation on multiple sites that determine activation, localization, and substrate specificity [2,27,28,29,30]. The goals of this review are to (1) review the overall structures and subfamilies of the PKCs and general activation mechanisms, (2) present an overview of the structure and unique regulation of PKCδ, and (3) describe the especially unique and critical roles of PKCδ in sepsis. We will focus on the specific phosphorylation sites of PKCδ that determine its critical role in cell signaling in inflammation. Finally, we will present genetic and pharmacological tools, as well as in vivo models, that can be used to examine the role of PKCδ in inflammation and sepsis, and how emerging tools such as microfluidics can be useful in such explorations. 

**Figure 1:**
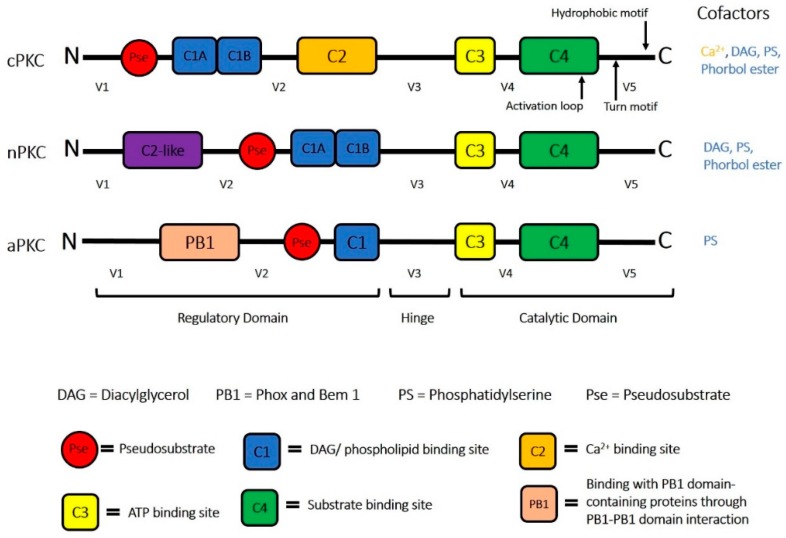
Structure of the three main classes of Protein Kinase C (PKC)s along with their respective activators. The hinge domain separates the regulatory domain and the catalytic domain. The regulatory domain contains: the pseudosubstrate (binds to C4 when not activated) for keeping the enzyme inactive; the C1 domain (including C1A and C1B) for DAG/PS/phorbol ester binding for cPKCs and nPKCs; the C2 domain for Ca^2+^ binding; the C2-like domain for nPKC spatial distribution; and the C1 domain (in aPKCs) for PS binding. The catalytic domain contains the C3 domain for ATP binding and C4 domain for substrate/pseudosubstrate binding.

**Figure 2:**
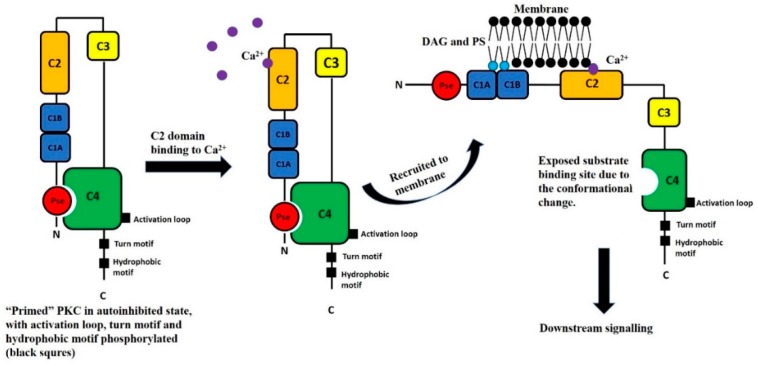
Schematic drawing of the activation steps of cPKCs. Following the three distinct phosphorylations at the activation loop, the turn motif, and the hydrophobic motif (for example, in human PKC β-II, corresponding to threonine 500, serine 641, and threonine 6601, respectively), PKCs are released into the cytosol, but with the pseudosubstrate occupying the substrate-binding site. Binding to Ca^2+^, PS, and DAG results in membrane translocation and subsequent conformational change, which releases the pseudosubstrate from the substrate-binding site.

The different PKCs share several common structural features (Figure 1). The catalytic domain, located at the C-terminus, contains the ATP binding site, as well as the substrate binding sites [2,5,31]. A hinge region connects the catalytic domain to the regulatory domain, which is a domain that regulates the activation state of the kinase through a pseudosubstrate region. The pseudosubstrate region is a substrate-mimicking short amino acid sequence that binds the substrate-binding cavity in the catalytic domain, rendering the enzyme inactive (Figure 2). PKCs contain several conserved membrane-targeting modules that are located in the regulatory domain (C1 and C2) and the catalytic domain (C3 and C4) [2]. 

The C1 region in the regulatory domain also contains the pseudosubstrate region that controls PKC activity. The C1 domain is also the binding site for DAG and PS, critical cofactors in cPKC and nPKC activation [5,32,33,34], as well as the non-hydrolysable, non-physiological analogues, phorbol esters. DAG is the product of the hydrolysis of the phospholipid phosphatidylinositol 4,5-biphosphate (PIP_2_) by phospholipase C (PLC), which yields inositol triphosphate (IP_3_) and DAG [8,27,35]. IP_3_ in turn activates signaling pathways that elevate intracellular Ca^2+^ levels and thereby activate cPKCs [27]. For cPKCs and nPKCs, the DAG-mediated activation is initiated by the docking of DAG/PS to the two cysteine-rich regions (C1A and C1B) in the C1 domain. This docking event weakens the interaction of an inhibitory pseudo-substrate domain with the C-terminus catalytic core and recruits cPKCs and nPKCs to the membrane compartment [36]. cPKCs are also regulated by changes in cytosolic Ca^2+^ concentrations. The C2 domain is a critical Ca^2+^-sensing membrane-targeting module in cPKCs [5]. The C2 domain in cPKCs binds two or three calcium ions [37,38,39] and facilitates the docking of cPKCs to the plasma membrane. In nPKCs, the C2-like domain lacks one or more of the conserved aspartate residues required for Ca^2+^ binding, and these isoforms are activated by DAG/PS in the absence of Ca^2+^ [5,29]. The function of the C2-like domain in nPKCs remains unclear. It is speculated that the C2-like domain is involved in the control of the nPKC spatial distribution via protein-protein interactions [31]. aPKCs, on the other hand, lack the C2 domain and have an incomplete C1 domain. Thus, aPKCs are Ca^2+^-insensitive, and do not respond to DAG. aPKCs are activated through the Phox and Bem 1 (PB1) domain, which is a protein interaction module that mediates aPKCs interactions with other PB1domain-containing scaffolding proteins and phospholipid co-factors such as PS [4,5,8,40,41]. The C3 and C4 domains form the ATP- and substrate-binding components, respectively, of the kinase core [34]. 

**Figure 3:**
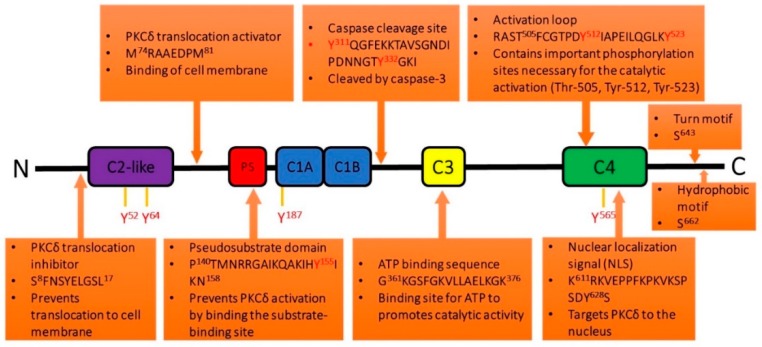
Important amino acid sequences (activators, inhibitors, regulatory signals) and tyrosine phosphorylation sites on PKCδ. Adapted from Malavez et al., 2009 [27].

PKCs also contain five variable regions, which are poorly conserved across the different PKCs [5]. For example, in PKCδ, the V1 region contains the translocation inhibitor site; V2 contains the translocation activation site; V3 (at the hinge region) contains serine phosphorylation sites at 299, 302, 304, and tyrosine 311 and 322 phosphorylation sites; V4 contains the ATP binding sequence; and V5 contains the turn and hydrophobic motifs, as well as serine 643 and 662 phosphorylation sites (Figure 3) [5]. 

In order for these allosteric interactions to occur, however, PKCs must first be properly folded and in the correct conformation permissive for catalytic action (Figure 2). This is contingent upon phosphorylation of the catalytic region, at the activation loop, the turn motif, and the hydrophobic motif [42]. First, PKCs are phosphorylated on the activation loop by phosphoinositide-dependent kinase, PDK-1, which functions as a switch to elicit the other two phosphorylations. Next, the turn motif and hydrophobic motif are autophosphorylated. After the three “priming” phosphorylation steps, the kinase is mature and released to the cytosol and is thus ready to respond to second messengers. It is worth noting that the activation loop phosphorylation is not required for the entire regulation process. Once the first step of phosphorylation is completed, the activation loop may be dephosphorylated [42].

Though the binding of DAG does not lead to a significant conformational change, it dramatically alters the surface properties of the kinase to create a hydrophobic surface for tight membrane binding. After binding to the membrane, the interaction of the C1 domain and the membrane leads to a conformational change that releases the pseudosubstrate from the substrate-binding site (Figure 2). This process readies the kinase to phosphorylate other proteins for downstream signaling. In the resting state, the pseudosubstrate of the regulatory domain occupies the substrate-binding site in the catalytic domain and maintains the enzyme in an inactive conformation. 

## 2. PKCδ and Its Unique Role in Health and Disease 

We have identified PKCδ as an import regulator of the inflammatory response in sepsis [8,19,22,43,44,45,46,47]. Multiple cell types express PKCδ and proinflammatory mediators involved in the septic response activate this kinase [44,48]. Importantly, PKCδ regulates neutrophil and endothelial proinflammatory signaling [22,46,47]. In neutrophils, PKCδ regulates inflammatory signaling, activation of the transcription factor NF-κB and proinflammatory gene expression, secretion of cytokines/chemokines, and reactive oxygen species (ROS) production [22,46]. In endothelial cells, PKCδ is involved in NF-κB activation, adhesion molecule expression, the release of inflammatory mediators important in neutrophil transmigration, and regulation of endothelial cell permeability [23,47]. Thus, PKCδ is an important signaling element in the regulation of neutrophil-endothelial crosstalk, neutrophil adherence/rolling/migration, and vascular endothelial damage [8,19,21,22,23,46,47].

### 2.1. PKCδ Activation

PKCδ, unlike other members of the PKC family, is unique in its regulation by tyrosine phosphorylation, activation mechanisms, and multiple subcellular targets [2,27].

#### 2.1.1. PKCδ Phosphorylation 

PKCδ activity is regulated by phosphorylation patterns, subcellular translocation, and cleavage in a context-dependent manner [2,29]. The three main conserved threonine and serine phosphorylation sites for PKCδ are Threonine-505 (Thr-505, activation loop), Serine-643 (Ser-643, turn motif), and Serine-662 (Ser-662, hydrophobic motif) [27]. However, PKCδ retains little phosphorylation in the activation loop (Thr-505) in many cell types [27]. Unlike other PKCs, mutations of Thr-505 to Alanine in PKCδ do not affect catalytic activity, but may be important for enzyme stability [27,49]. In general, phosphorylation of Ser-643 and Ser-662 is necessary for PKCδ catalytic activation and Thr-505 phosphorylation can enhance the catalytic activity of PKCδ [27,50,51,52]. 

Unlike serine and threonine phosphorylation, tyrosine phosphorylation is not conserved among the different PKCs and PKCδ activation is uniquely regulated by tyrosine phosphorylation patterns (Figure 3) [27,29,53]. Human PKCδ contains 20 tyrosine residues (19 for mice and 21 for rat) [29], and includes phosphorylation sites in the regulatory domain (Tyr-52, Tyr-64, Tyr-155, and Tyr-187), the hinge region (Tyr-311 and Tyr-332), and the catalytic domain (Tyr-505, Tyr-512, and Tyr-523) [27]. Tyrosine phosphorylation of the catalytic domain increases PKCδ activity, while tyrosine phosphorylation in the regulatory domain influences cellular actions rather than catalytic competence [2,12].

Two important tyrosine phosphorylation sites are PKCδ Tyr-155 and PKCδ Tyr-311, which are critical phosphorylation sites associated with PKCδ-mediated proinflammatory signaling and the initiation of cytotoxic/apoptotic pathways [54,55,56]. Phosphorylation of PKC*δ* at Tyr-155 and Tyr-311 is required for nuclear translocation and enzyme cleavage [24,54,55]. Tyr-155 is located between the regulatory domain pseudo-substrate motif and the C1A domain and regulates apoptosis and gene expression [29,30,57]. PKCδ phosphorylation at Tyr-311, located in the hinge region, causes a conformational change that reveals the caspase cleavage site [29]. Our recent studies demonstrate that PKCδ Tyr-155 and PKCδ Tyr-311 are phosphorylated during sepsis and play key roles in sepsis-induced lung injury, the regulation of microvascular endothelium barrier function, and neutrophil-endothelial cell interactions (See Section 2.2.3 and Section 2.2.4) [21,24]. Tyr-155 phosphorylation is also significant in cell apoptosis; mutations of this site increase cell proliferation in response to PMA [27,30]. Tyr-187 is a major phosphorylation site in response to PMA, PDGF, and etoposide, but does not appear to affect PKCδ kinase activity [12,58]. Tyr-187 and Tyr-64 are important phosphorylation sites for etoposide-induced apoptosis [58]. Tyr-52 is unique to PKCδ and located at the C2 domain [29,59]. Lyn, a member of the Src family kinases, phosphorylates PKCδ on Tyr-52, and this phosphorylated tyrosine residue then serves as a docking site for the SH2 (Src homology 2) domain of Lyn and reciprocal phosphorylation [60,61,62]. Tyr-52 is also phosphorylated in response to IgE in leukemia cells, and IgE-stimulated PKCδ phosphorylation reduces its activity to certain substrates, suggesting that PKCδ tyrosine phosphorylation may be important in substrate recognition [58]. Tyr-311, Tyr-332, and Tyr-512 are important phosphorylation sites for kinase activation and subcellular localization [12,27,58]. In addition, PKCδ Tyr-332 phosphorylation creates a docking site for Shc [12]. 

In addition to identification of the different functions and mechanisms of the individual tyrosine phosphorylation sites of PKCδ, the identification of PKCδ-specific substrates is also important to understand how this kinase regulates cellular function. Table 1 summarizes proteins identified as PKCδ substrates. For example, PKCδ preserves homeostasis by phosphorylating plasma membrane calcium ATPase (PMCA) that helps regulate calcium levels in the skin [27,63,64]. PKCδ phosphorylates caspase-3 in human monocytes, which promotes the apoptotic activity of caspase-3 both in vitro and in vivo [65]. PKCδ also phosphorylates the p52Shc protein at Ser-29 (when under oxidative stress), p66Shc at Ser-138 (ERK activation), and Heat Shock Protein 25 (HSP25) through binding at the V5 region, leading to the inhibition of apoptosis [29,66,67,68]. Additional substrates of PKCδ have been discovered with the aid of PKCδ inhibitors and activators, such as cytoskeleton proteins [28], the myristoylated alanine-rich C-kinase substrate (MARCKS) [28,69], troponin [28,70], the nuclear protein DNA-dependent protein kinase [28,71], and pyruvate dehydrogenase (a mitochondrial enzyme) [28,72]. Thus, PKCδ has a myriad of phosphorylation targets, and further studies are required to determine the targets of PKCδ phosphorylation in specific cells and in various disease conditions, particularly in sepsis.

#### 2.1.2. PKCδ Translocation and Subcellular Localization

PKCδ has been classically known to move from the cytosol to the plasma membrane upon activation into a mature, catalytically competent form. However, recent investigations have revealed that PKCδ can move to several subcellular compartments, including mitochondria, endoplasmic reticulum (ER), Golgi apparatus, nuclei, and caveolae [48,73,74,75,76]. This translocation of PKCδ is mediated by tyrosine phosphorylation [29]. In cardiomyocytes, PKCδ moves from the nucleus to focal contacts and cytoskeleton and around the nucleus [48]. PMA can enhance the movement of PKCδ to caveolae, leading to increased ERK activity [73]. PKCδ in its tyrosine phosphorylated form can also accumulate in the soluble portion of hydrogen peroxide-treated cardiomyocytes and, in itself, can act as a lipid-independent kinase [29]. PKCδ can transiently translocate to the ER following ER stress and binds to Abl (a tyrosine kinase) [74]. After briefly translocating to the ER, PKCδ then accumulates in the mitochondria, inducing apoptosis [74]. In human leukemia cells, ceramide release is caused by TNF-α-initiated apoptosis and the translocation of PKCδ from the plasma membrane to Golgi apparatus [75]. In glioma cells, PKCδ was found to induce apoptosis when targeted to the cytoplasm, nucleus, and mitochondria, whereas the ER translocation protected the cells from TNF-ligand-induced cell death [76]. Overall, there is no uniform pattern of PKCδ tyrosine phosphorylation and it is becoming more evident that the precise configuration of tyrosine phosphorylation depends on the stimulus that dictates the functional properties of the enzyme and its subcellular location. For example, in platelets, thrombin-induced Tyr-311 phosphorylation on PKCδ occurs subsequent to Thr-505 phosphorylation, while ADP-induced Tyr-311 phosphorylation does not appear to require the threonine phosphorylation [77]. In a rodent model of sepsis, pulmonary PKCδ is phosphorylated on both Tyr-155 and Tyr-311, resulting in PKCδ nuclear translocation and PKCδ cleavage [24]. Thus, PKCδ activation is stimulus-dependent and cell type-specific. 

### 2.2. PKCδ in Inflammatory Diseases

We identified PKCδ as a critical regulator of the inflammatory response in sepsis and an important signal transducer of multiple signaling pathways [8,19,20,21,22,23,25,43,44,45,46,47]. PKCδ is activated by inflammatory mediators involved in sepsis, including pathogen associated molecular patterns (PAMPs) such as LPS and the bacterial peptide fMLP, as well as the proinflammatory cytokines TNF and IL-1 [44,48,99]. Moreover, PKCδ is activated in multiple cell types and organs in animal models of sepsis [19,47]. Key to sepsis-induced tissue damage is the excessive migration of activated neutrophils across the vascular endothelium [100,101,102,103]. Studies with PKCδ^−/−^ mice and PKCδ inhibitors indicate a role for PKCδ in regulating neutrophil trafficking to the lung in response to inflammation triggered by bacterial sepsis, asbestos, stroke/reperfusion injury, LPS, or pancreatitis [19,20,24,47,104,105,106,107].

#### 2.2.1. Role of PKCδ in Sepsis—Animal Studies

During sepsis, systemic inflammation leads to increased adhesion molecule expression on neutrophils and endothelial cells, resulting in increased neutrophil-endothelial cell interaction, vascular endothelial damage, and organ dysfunction [108,109,110]. While neutrophils are critical to host defense, neutrophil dysregulation has a critical role in the early course of organ damage through the release of proteases, neutrophil extracellular traps (NETs), and oxygen radicals. Increased neutrophil recruitment in sepsis is associated with tissue damage, multiple organ dysfunction syndrome (MODS), and increased mortality [100,101,102,111]. 

Using a clinically relevant rodent model of polymicrobial sepsis induced by cecal ligation and puncture (CLP), we found significant lung injury within 24 hrs post CLP surgery, including increased neutrophil accumulation in lung tissue, pulmonary permeability, tissue edema, altered lung mechanics, and disrupted lung architecture [19,20,24,47]. In this sepsis model, we found PKCδ activation and phosphorylation on multiple sites, including Ser-643/676, Thr-505, Tyr-155, and Tyr-311 [19,24,25]. 

To examine the regulatory role of PKCδ in sepsis, we employed a selective peptide inhibitor developed by Mochly-Rosen’s group [13]. This inhibitory peptide is derived from the first unique region (V1) of PKCδ (SFNSYELGSL; amino acid residues 8 to 17, see Figure 3), targets docking domains, and prevents translocation and substrate interaction [13]. This inhibitor targets the regulatory domain of PKCδ, but not the ATP binding site, so it is more specific than previously described PKCδ inhibitors such as rottlerin. Rottlerin has been shown to be a mitochondria uncoupler and, in recent years, has been shown to modulate biological and biochemical events in a PKCδ-independent manner [112,113]. This PKCδ peptide inhibitor is coupled to a membrane permeant TAT peptide (YGRKKRRQRRR) that allows safe and effective intracellular delivery into target cells in vitro and in vivo [13,19,22,46,114,115]. Administration of the PKCδ peptide inhibitor in our animal model of sepsis decreased pulmonary PKCδ phosphorylation, attenuated lung injury, and improved gas exchange, indicating that PKCδ inhibition is lung protective in sepsis [19,20,24,47]. 

Further studies demonstrated that PKCδ inhibition reduced neutrophil influx into multiple organs, including the lung, kidney, and brain [20,24,25,47]. The vascular endothelium is involved in the pathogenesis of sepsis and is an active participant in the dynamic process of recruitment and activation of neutrophils through the production of chemokines/cytokines and expression of adhesion molecules [100,116,117,118,119]. ICAM-1 and VCAM-1 are crucial vascular endothelial cell adhesion molecules involved in neutrophil recruitment and are up-regulated by proinflammatory cytokines released during sepsis [120]; their expression was, however, attenuated by the administration of the PKCδ peptide inhibitor [47]. These studies suggest a link among PKCδ activity, the upregulation of adhesion molecules, and increased neutrophil migration in the injured lung. PKCδ was also activated in the brain in this sepsis model, resulting in increased PKCδ Ser-643 phosphorylation and membrane translocation [25]. PKCδ activation was associated with increased blood brain barrier (BBB) permeability that was attenuated by administration of the PKCδ peptide inhibitor [25]. 

#### 2.2.2. Role of PKCδ in Neutrophil-Endothelial Cell Interactions—In Vitro Studies Using Microfluidics-Based Biomimetic Assays

**Figure 4:**
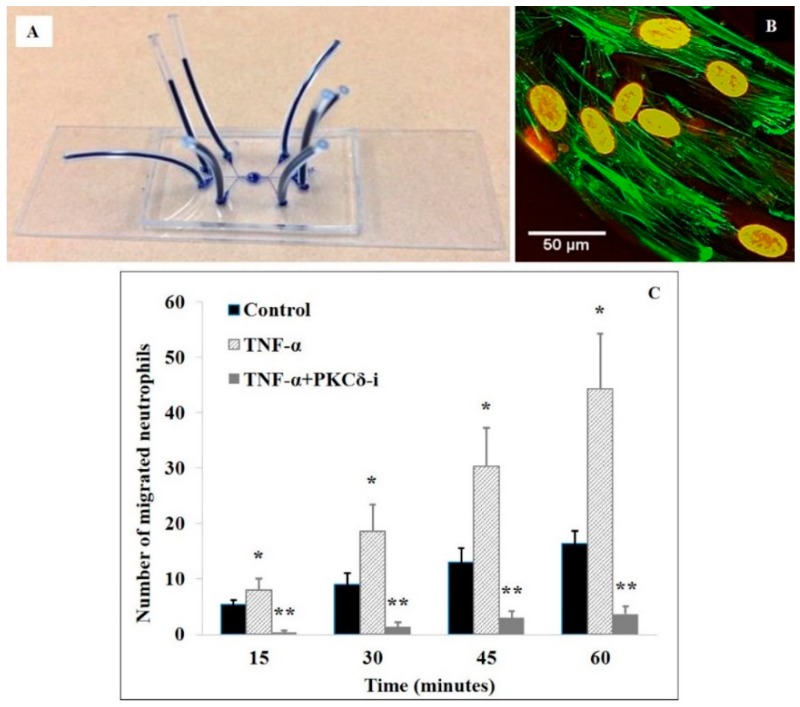
Microfluidic-based in vitro assay for studying the role of PKCδ in regulating neutrophil-endothelial cell interaction. (**A**) The assay is manufactured by soft lithography on polydimethylsiloxane (PDMS) and assembled on a microscope glass slide with plastic tubes (dark blue) allowing access to individual vascular channels and the tissue compartment. (**B**) 3D reconstruction of confocal images of human brain microvascular endothelial cells (HBMVEC) stained for F-actin with fluorescently labelled phalloidin (green) and for cell nuclei with Draq 5 (red) after 72 hrs of flow culture (0.1 μL/min). (**C**) PKCδ inhibition with a PKCδ-TAT peptide inhibitor (PKCδ-i) reduces neutrophil migration across activated HBMVEC. Data are presented as mean ± SEM (*n* = 3). ** *p* < 0.01, * *p* < 0.05 compared to the other two groups by *ANOVA* with Tukey-Kramer post-hoc. Reprinted with permission from Tang et al., 2018 [25].

Microfluidic systems provide a unique opportunity to explore in vitro the role of PKCδ in regulating neutrophil-endothelial cell interaction under physiologically realistic conditions [21,23,25,26]. Our group developed a novel microfluidic system (Figure 4) that resolves and facilitates the real-time assessment of individual steps, including the rolling, firm arrest, spreading, and migration of neutrophils into the extra-vascular tissue space in a single system. A Geographic Information System (GIS) approach [121] was used to digitize microvascular networks for the subsequent generation of synthetic microvascular networks using soft-lithography processes to develop a bioinspired microfluidic assay (bMFA). This bMFA was based on microvascular network morphologies obtained from in vivo animal data [122,123,124,125,126]. This microfluidic assay consists of vascular channels in communication with a tissue compartment filled with chemoattractants via a porous barrier. Neutrophils circulate in the vascular channels on a monolayer of activated endothelial cells under physiologic shear conditions. 

In the bMFA, TNF-α activated human endothelial cells and upregulated the expression of the adhesion molecules and neutrophil adhesion to them [23]. Neutrophil adhesion was shear-rate dependent, with increased adhesion in vessels with lower shear rates and near bifurcations [23], and endothelial cells treated with the PKCδ inhibitor showed significantly decreased neutrophil adhesion and migration, consistent with our in vivo observations [21,23]. Mechanistic studies demonstrated that PKCδ regulates expression of the adhesion molecules E-selectin and ICAM-1. PKCδ is also an important regulator of endothelial cell permeability, and PKCδ inhibition attenuated TNFα-mediated endothelial cell permeability and decreased transendothelial electrical resistance (TEER) [25]. Similar changes in human brain microvascular endothelial cell permeability were obtained by employing a novel blood-brain-barrier (BBB) on-a-chip (B^3^C) microfluidic system [25] (Figure 4). Thus, PKCδ plays a key role in the regulation of proinflammatory signaling controlling the activation and recruitment of neutrophils and in regulating endothelial permeability, TEER, and tight junction protein expression [8,19,20,21,23,24,25,47].

PKCδ is also an important regulator of neutrophil-endothelial cell interactions in radiation-induced inflammation and vascular injury. Studies from our group and others have shown that the exposure of tissue to ionizing radiation (IR) causes an increase in leukocyte infiltration across endothelium and loss of barrier function [127,128,129,130]. Key to radiation-induced tissue damage is the excessive migration of activated neutrophils across the vascular endothelium [131,132]. In studies with human endothelial cells, we found that exposure to X-ray radiation (0.5–5 Gy) activated PKCδ through phosphorylation (Ser-643) and translocation to membrane fraction [26]. Using our bMFA, we showed that PKCδ regulates radiation-induced neutrophil-endothelial cell interaction and endothelial cell function, and that PKCδ inhibition dramatically attenuated IR-induced endothelium permeability and significantly decreased neutrophil migration across IR treated endothelial cells [26]. Moreover, neutrophil adhesion to irradiated endothelial cells was significantly decreased after PKCδ inhibition in a flow-dependent manner. PKCδ inhibition downregulated the IR-induced overexpression of P-selectin, ICAM-1, and VCAM-1, but not of E-selectin. Thus, PKCδ is an important regulator of neutrophil-endothelial cell interaction post-IR exposure.

#### 2.2.3. PKCδ Phosphorylation in Sepsis and Inflammation—In Vivo Studies

**Figure 5:**
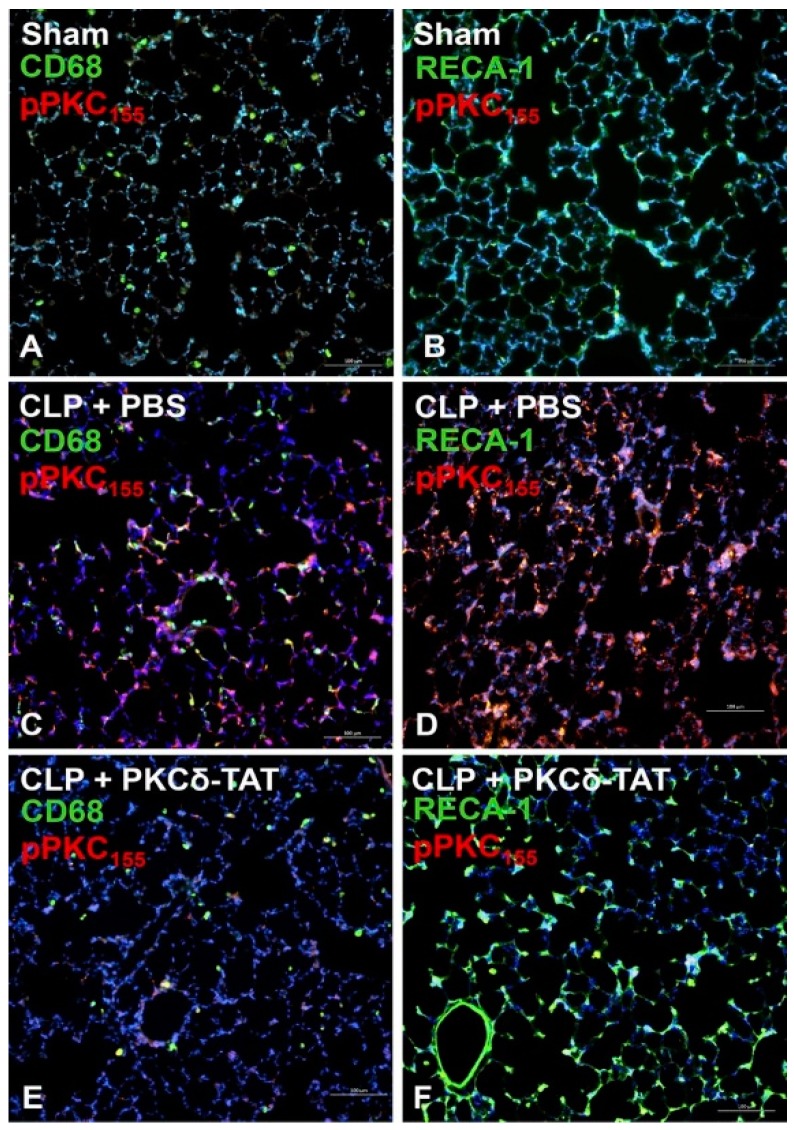
Immunohistochemical analysis of PKCδ phosphorylation at tyrosine 155 (pPKCδ_155_; red) in lung tissue sections at 24 h post-surgery of sham-operated animals (Sham) (**A**,**B**) and CLP-operated animals that received 200 μg/kg PKCδ-TAT (CLP + PKCδ-TAT) (**E**,**F**) or a similar volume of PBS vehicle only (CLP + PBS) (**C**,**D**) immediately following surgery. (**A**,**C**,**E**) Tissue sections were also stained for CD68 (green), a marker for the cells of the macrophage lineage. Yellow/orange indicates co-localization of pPKCδ_155_ and CD68. (**B**,**D**,**F**) Tissue sections were also stained for rat endothelial cell antigen-1 (RECA-1; green), a marker for rat endothelial cells. Yellow/orange indicates co-localization of pPKCδ_155_ and RECA-1. All scale bars = 100 microns. Reprinted with permission from Mondrinos et al., 2015 [24].

Our in vivo studies demonstrated that sepsis triggered significant tyrosine phosphorylation of PKCδ [24]. Sepsis-induced lung injury was characterized by the phosphorylation of PKCδ at Tyr-311 throughout the distal lung, which is consistent with the finding that Tyr-311 is a critical phosphorylation site in the context of vascular inflammation [24,133]. Of particular interest, pulmonary endothelial cells, in contrast to pulmonary macrophages and epithelial cells, were the primary cell type exhibiting Tyr-155 phosphorylation in response to sepsis (Figure 5) [24]. This is a key observation, as in sepsis, pulmonary endothelium contains the first cells in the lung to encounter systemic proinflammatory mediators, making them the frontline inflammatory responders in systemic inflammation. 

To interrogate the role of Tyr-155 phosphorylation in sepsis-induced lung injury and neutrophil recruitment to the lungs, PKCδ knock-in (KI) mice were produced where PKCδ Tyr-155 was mutated to phenylalanine (PKCδY155F KI mice) [21]. Compared to wild-type (WT) septic mice, there was a significant decrease in neutrophil recruitment to the lungs in PKCδY155F KI septic mice, indicating an important role for Tyr-155 phosphorylation in regulating proinflammatory activity during sepsis [21].

#### 2.2.4. PKCδ Phosphorylation in Sepsis and Inflammation—In Vitro Studies

To investigate the role of PKCδ Tyr-155 phosphorylation in neutrophil superoxide anion (O_2_^−^) generation, bone marrow neutrophils were isolated from PKCδY155F KI mice [21]. PKCδY155F bone marrow neutrophil O_2_^−^ production in response to fMLP or TNFα activation was significantly decreased compared to WT mice. Decreased O_2_^−^ production was stimulus-dependent as PMA-mediated O_2_^−^ generation was not affected. Formation of neutrophil extracellular traps (NETs) from PKCδY155F KI mice was also attenuated in response to IL-1 or TNF as compared to WT mice. Hence, PKCδ is an important regulator of O_2_^−^ and NETs release, and PKCδ Tyr-155 is a key phosphorylation site regulating proinflammatory signaling controlling neutrophil activation [21]. 

To investigate further the role of PKCδ Tyr-155 phosphorylation in neutrophil-endothelial interaction in inflammation, we employed the bMFA to examine endothelial cell permeability and neutrophil migration [21]. Our studies demonstrate that the Tyr-155 phosphorylation site is a critical regulator of endothelium barrier function, neutrophil adhesion, and neutrophil transmigration. Consistent with our previous findings [21,23], PKCδ was found to play a more significant role in regulating the migration of neutrophils across endothelial cells as opposed to their adhesion to endothelial cells. Overall, these findings indicate that regulating PKCδ activity may provide novel therapeutic strategies for treating inflammation. 

## 3. Concluding Remarks

The Protein Kinase C superfamily consists of multiple isoforms with separate and overlapping cellular and physiological functions that contribute to health and disease. Among them, PKCδ has a unique tyrosine phosphorylation pattern that diminishes or enhances biological processes such as neutrophil and platelet adhesion, migration, and adhesion molecule expression. Furthermore, the inhibition of PKCδ may offer a therapeutic pathway for reducing neutrophil-mediated organ damage in inflammatory diseases. Emerging in vitro methods (e.g., microfluidic platforms) provide unique perspectives for delineating biological mechanisms in a physiologically relevant environment prior to observation and study in animal models or clinical settings, reducing drug development costs and providing more precise and personalized diagnostic/treatment methods. 

## Figures and Tables

**Table 1:** PKCδ substrates and functions. Adapted from Steinberg 2004 [29].

Substrate	Effects
c-Abl	Increased activity [78,79]
SFKs	Variable [80]
SHPTP1 (protein tyrosine phosphatase) (SHP1)	Decreased phosphatase activity [81]
RasGRP	Uncertain [82]
Protein tyrosine phosphatase PTPα	Increased phosphatase activity [83]
PKCε (hydrophobic motif)	Yields release from membranes [50]
STAT1 (Ser-727)	Interferon gene expression [84]
STAT3 (Ser-727)	Reduced DNA binding and transcription [85]
p300	HAT activity lowered, decreased transcriptional function [86]
14-3-3	Interfere with 14-3-3 polymerization and interactions with partners [87]
gp130	Increased gp130-STAT3 interaction [88]
p47(pbox) unit of NADPH	Activity enhancement [89]
β4-integrin	Cell-laminin attachment decreases [90]
Caspase-3	Promote the apoptotic activity of caspase-3 in monocytes both in vitro and in vivo [65]
MARCKS	Cell attachment and spreading in skeletal muscle cells [69]
M2 Pyruvate Kinase	Tumor metabolism; uncertain [91]
Heat Shock Protein 27 (HSP27)	Protein chaperone, antioxidant, apoptosis inhibition [92]
Plasma membrane calcium ATPase (PMCA)	Regulate calcium levels in skin [30,65]
Heat Shock Protein 25	Inhibition of apoptosis [92]
p52Shc protein	Positively regulates H2O2-induced ERK activation [67]
p66Shc protein	Negatively regulates H2O2-induced ERK activation [67]
Troponin	Decreased Calcium sensitivity of actomyosin [70]
Pyruvate Dehydrogenase Kinase	Inhibition of PDH resulting in necrosis and blocking ATP regeneration [71]
DNA-dependent protein kinase	Inhibition of p53 phosphorylation [93]
Bcl-2-associated death promoter (BAD)	Promotes apoptosis post-reperfusion after cardiac ischemia [94]
Dynamin-related protein 1 (Drp1)	Induction of mitochondrial fission and dysfunction following cardiac ischemia [17]
Glyceraldehyde-3-phosphoate dehydrogenase (GADPH)	Removal of injured mitochondria following ischemic damage [95]
PLS3	Higher phospholipid movement [96]
DNA-PK	Increase apoptosis due to malfunctional DNA [93]
Lamin B	Apoptosis [97]
hRad4	Increased hRad9-Bcl-2 interactions/apoptosis [98]
p73β(Ser-289)	p73β activation; apoptosis [66]
